# Lifestyle Matters: Effects of Habitual Physical Activity on Driving Skills in Older Age

**DOI:** 10.3390/brainsci12050608

**Published:** 2022-05-06

**Authors:** Evrim Gökçe, Robert Stojan, Melanie Mack, Otmar Bock, Claudia Voelcker-Rehage

**Affiliations:** 1 Department of Neuromotor Behavior and Exercise, Institute of Sport and Exercise Sciences, University of Münster, Wilhelm-Schickard-Straße 8, 48149 Münster, Germany; robert.stojan@uni-muenster.de (R.S.); melanie.mack@uni-muenster.de (M.M.); 2 Sports Health Rehabilitation Laboratory, Ankara City Hospital, Ankara 06800, Turkey; 3 Institute of Exercise Training and Sport Informatics, German Sport University, Am Sportpark Muengersdorf 6, 50927 Cologne, Germany; bock@dshs-koeln.de

**Keywords:** aging, car-driving, multitasking, driving simulator, physical activity, fitness, old-old, young-old

## Abstract

Research on multitasking driving has suggested age-related deterioration in driving performance. It has been shown that physical and cognitive functioning, which are related to driving performance and decline with aging, are positively associated with physical activity behavior. This study aimed to explore whether driving performance decline becomes severe with advancing age and whether physical activity behavior modifies age-related deterioration in driving performance. A total of one hundred forty-one healthy adults were categorized into three groups based on their age; old-old (74.21 ± 2.33 years), young-old (66.53 ± 1.50 years), and young adults (23.25 ± 2.82 years). Participants completed a realistic multitasking driving task. Physical activity and cardiorespiratory fitness levels were evaluated. Older groups drove more slowly and laterally than young adults, and old-old adults drove slower than young-old ones across the whole driving course. Physical activity level did not interact with the aging effect on driving performance, whereas cardiovascular fitness interacted. Higher-fitness young-old and young adults drove faster than higher-fitness old-old adults. Higher-fitness old adults drove more laterally than higher-fitness young adults. The present study demonstrated a gradual decline in driving performance in old adults, and cardiorespiratory fitness interacted with the aging effect on driving performance. Future research on the interaction of aging and physical activity behavior on driving performance in different age groups is of great value and may help deepen our knowledge.

## 1. Introduction

In everyday life, performing complex motor and cognitive tasks simultaneously (multitasking) is required to sustain functionality and independent living. Driving a car is a typical activity of daily life in which the simultaneous performance of motor tasks such as steering, braking, accelerating and cognitive tasks such as observing traffic flow, recognizing traffic signs, or driving directions is indispensable. This simultaneity demands the interaction of motor, sensory and cognitive functions [1]. In most cases, driving even includes additional (secondary) tasks like mobile texting, listening to a podcast, or talking to a fellow passenger. These additional tasks require motor and sensory resources alongside driving the vehicle in a changing environment. Performing cognitively demanding secondary tasks might reveal conflicts in distributing the available cognitive resources to the driving and secondary task and thereby increase the relative risk of a crash [2,3].

Many older adults report that driving brings them freedom, competent control of their lives, and safety since they conceive public transport as riskier [4]. In addition, driving seems to be an important component of independence and emotional well-being in older adults [5]. However, the growing proportion of drivers aged ≥ 65 years due to the population aging has been associated with an increased risk of fatal accidents [6,7] and involvement in motor vehicle collisions [8]. Motor vehicle accident data which is adjusted for travel distance showed an exponential increase above the age of 75 [9]. Particularly, those aged ≥ 75 seem to be involved in a higher number of road crashes and mortality rates than young-old and young adults [10,11], indicating that driving performance declines (even further) with increasing age. Thus, continuing driving until a high age and driving safety are important topics for the older driver and society. 

It is widely known that motor, sensory and cognitive skills, particularly executive functions, which all affect driving performance, tend to decline with increasing age [12,13,14,15,16]; thus, driving becomes a more challenging task for older adults [17,18]. For example, studies comparing old and young adults revealed significant differences in abilities and skills inevitable for driving performance such as executive functioning, motor programming, processing speed, or working memory [19,20,21,22,23,24,25,26]. Accordingly, the literature indicates that driving errors engaging older drivers are likely due to declining cognitive abilities (e.g., cognitive processing speed, sustained attention) [9,27,28,29]. 

Age differences in favor of young adults have been found in multitask driving for reaction time [30], driving safety error [18,28,31,32,33], and crash rate [24,28]. In all these experiments, adults between 60 and 80 years of age have been included, but this age range was not further subdivided. Age ranges for the elderly groups were often extensive in most of the studies, comprising 10 to 25 years. Some studies did not specify the age range of their older drivers [28,31,32,34] while others specified a very wide age range [30,33]. However, aging literature indicates that some perceptual, cognitive, and motor skills (e.g., perception, temporal information processing, executive functioning, motor coordination) related to driving ability gradually deteriorate across the adult life span [20,35,36,37,38,39]. Moreover, as mentioned above, the number of fatal crashes per distance increases with advancing age. Put together, it may be important to distinguish between young-old and old-old participants when assessing age-related differences in driving performance. Old-old adults might perform worse and might therefore be more likely to apply different cognitive strategies than young-old adults to deal with increased task loads. Mixing those two groups may obscure the possible consequences of advancing age, as it will average the potential effects.

Several factors already have been demonstrated to influence driving performance in older adults, including driving practice [40], sex [31], and living habits [41]. One other factor that might influence driving behavior, but has been neglected so far, are physical activity behavior and cardiovascular fitness. Physical activity is defined as any bodily movement produced by skeletal muscles requiring energy expenditure, including during leisure time, transport, or work [42]. Many studies report that physical activity level is a determinant of physical functioning in older adults [43,44,45]. Even though driving may seem like a passive activity where drivers are comfortably seated at first glance, it has been shown that driving difficulties or the risk of crashing are related to physical functioning [46,47,48]. In this context, it was already demonstrated that physical training increased driving performance in physically impaired older drivers [49]. As a modifiable lifestyle determinant, physical activity level is also discussed as a possible moderating factor for the cognitive deterioration effects of aging [50,51,52,53,54]. It was shown that, for example, daily activities involving physical exertion are associated with reduced cognitive loss in older adults [50] and engaging in physical activity attenuates the risk of progressive cognitive decline [51]. Higher physical activity level has been associated with better global cognitive function in older adults [52]. Longitudinal studies showed that older adults who engage in physical activity have less cognitive decline over two to ten years of follow-up [53,54].

Related to physical activity, cardiorespiratory fitness (CRF) is suggested as a critical mechanism involved in the beneficial effects of physical activity on neurocognitive health [55,56,57,58]. However, typically physical activity level and CRF are only weakly correlated [59]. Better cognitive performance in healthy older adults has been also linked to higher CRF, especially executive functions that are vulnerable to age [60,61]. Research also demonstrated that both, physical activity level and CRF, seem to be positively associated with those cognitive and motor functions in older adults that are related to driving behavior, such as visuospatial accuracy [62], visual scanning and attention [63,64], spatial attention and executive control [65,66,67], or processing speed [68,69,70]. Considering that cognitive functions are indispensable for driving [71], the effect of CRF on cognition may also have an effect on driving performance, particularly under cognitively demanding conditions such as driving and concurrently performing a secondary task. Hence having a higher physical activity level and/or CRF might preserve driving performance in older adults, especially under higher cognitive load. However, few studies investigated the relationship between physical activity level and/or CRF and driving performance in healthy older adults [72,73]. A cross-sectional study showed that physical activity level was not a predictor of driving performance [72]. Conversely, one recent study demonstrated that cardiovascular fitness had a trend-level indirect effect on speed control during dual-task driving [73]. Brought together, understanding the possible effect of physical activity behavior and CRF on age-related driving performance loss might provide further insights and strategies to maintain driving skills even in demanding situations, e.g., while performing secondary tasks.

The primary aim of the present study was to investigate the development of driving performance decline in older adults and whether the severity of performance loss increases with advancing older age while performing concurrent subtasks in an ecologically valid driving scenario. Earlier studies compared young-old and old-old adults on measures such as hazard perception, change blindness, and gap judgments [35,74,75]. To the best of our knowledge, no previous research has investigated driving performance in different groups of older adults under cognitively demanding dual-tasking conditions by focusing on longitudinal and lateral control measures, the other critical components of road safety. We hypothesized that the negative effect of advancing age on driving performance would be demonstrated not only between young and older adults but also within the group of older adults, between young-old and old-old adults.

The second aim of this study was to understand whether physical activity behavior and/or CRF modulates the hypothesized age-related deterioration in driving performance. Considering higher levels of physical activity and/or CRF had been associated with healthy aging [76,77] and better cognitive performance [50,52]; more active/higher-fitness old adults might resist the deterioration effect of aging on driving performance. We hypothesized that a higher physical activity level and CRF might interact with the effects of aging on driving performance. This study goes beyond the previous literature by investigating the effect of physical activity level and CRF on driving performance in young-old and old-old adults rather than investigating a wide age range of old adults. We expected the positive association of higher physical activity level/CRF with driving performance would differ in age groups and would be more pronounced in the old-old group where age-related driving performance loss is most prominent.

## 2. Materials and Methods

### 2.1. The Study Design and Participants

This study was performed within the DFG (German Research Foundation) Priority Program SPP 1772 “Multitasking”. The project has two phases, and to test our hypothesis, data sets from both phases were combined. In total, 141 healthy males (n = 73) and females (n = 68) between 20 and 79 years of age were recruited via public advertising, including local/social media, and contacts with senior networks from German Sport University Cologne and Chemnitz University of Technology, Germany. Participants were divided into three groups based on their age; old-old (70–79 years, n = 46), young-old (65–69 years, n = 47), and young adults (20–30 years, n = 48). The demographic characteristics of the participants of each group are presented in Table 1.

Participants were screened for eligibility through a structured phone interview (10–15 min). The inclusion criteria obtained by self-report comprised

- regular driving history during the last six months (at least once per week)
- body mass index < 30
- no experience of driving simulator research
- good physical and mental health (stated by physician)
- normal or corrected to normal vision and hearing
- no history of neurological/psychological disorder
- no ongoing orthopedic disorder
- right-handedness (only second project phase)

Those who met the above inclusion criteria completed a set of screening tests. Hand preference was assessed by the “Edinburgh Handedness Inventory”, visual acuity by the “Freiburg Visual Acuity Test (version 3.9.0, cutoff: 20/60)”, and cognitive impairment by the “Mini-Mental State Examination” (cutoff: 27/30). Nobody had to be excluded because of these criteria. Education level was assessed by a demographic questionnaire. 

The study was approved by the ethics committee of the German Sport University, Cologne (Nr.: 27/2015) and Chemnitz University of Technology, Germany (Nr.: V-280-17-CVR-Multitasking-29062018). A written consent form was obtained from all participants in accordance with the Declaration of Helsinki.

### 2.2. Measures

#### 2.2.1. Physical Activity Level

##### Physical Activity Questionnaire

Physical activity level was assessed by an adapted version of the German version of the Baecke Physical Activity Questionnaire [78]. The adapted version of the questionnaire consists of sixteen questions in different domains of occupational, sports, and leisure time activities. There were additional questions that were not in the German adapted version, such as secondary sports practice, interruption periods for sports, the intensity of the weekly average physical activity, and the intensity of the engaged sports. Standing, sitting, and walking duration for non-sport context was asked for while working (young adults only) and in leisure time.

##### Data Analysis

Physical activity behavior was analyzed according to the guidelines of the Baecke questionnaire [78]. Each domain could receive one to five points, resulting in a total score ranging from three (minimum) to fifteen (maximum). The information gained from the questionnaire (the intensity, frequency, and duration of the activity) was then used to calculate a total metabolic equivalent (MET) score for each activity by representing the specific activities performed in various settings [79]. Summing the score of MET-hours per week of each activity presented the total physical activity level. For physical activity data, the MET score was calculated by multiplying the corresponding MET value of the activity by the time involved in this activity. Missing data (n = 2) were imputed by regression imputation.

#### 2.2.2. Cardiovascular Fitness

##### Peak Oxygen Uptake (VO_2_peak)

Cardiovascular fitness was assessed as a peak oxygen uptake estimate that reflects cardiac function and skeletal muscle mitochondrial capacity [80]. VO_2_peak (L/min) was estimated by a graded exercise test (spiroergometry, ZAN600 CPET, nSpire Health, Oberthulba, Germany) on a stationary bicycle (Lode Corival cpet, Groningen, The Netherlands) with a ramp protocol [81].

Before testing, participants were asked to avoid caffeine and alcohol intake for 12 h and any strenuous physical activity for 24 h. Participants were required to cycle between 60 and 80 revolutions per minute. During phase I of the project, participants cycled at 30 W initial load that gradually increased by 15 W (male) or 10 W (female) per minute. Participants of project phase II cycled with a 20 W (male) or 10 W (female) initial load that gradually increased by 20 W (male) or 15 W (female) per minute. Heart rate (ECG, recorded with a 10-lead ECG fully digital stress system; Kiss, GE Healthcare, Munich, Germany), breath-by-breath respiration (oxygen uptake (VO_2_), carbon dioxide output (VCO_2_)), the respiratory exchange ratio (VCO_2_/VO_2_), blood pressure (every 2 min), and wattage level were continuously assessed.

The total protocol lasted about 15–20 min, including a three-minute resting and a five-minute cool-down period (1 min initial load and 4 min no load). During cycling, the perceived exertion rate was evaluated using the Borg scale (6: “no exertion at all”, 20: “maximal exertion”) every 2 min. Test termination criteria were the presence of risk factors (e.g., chest pain, dizziness, cardiac arrhythmia, blood pressure >230/115 mmHg, and other abnormalities), volitional exhaustion, or reaching a respiratory exchange ratio of >1.05 for about 30 s or >1.10 instantly. The assessment was managed under the supervision of an expert sports scientist.

##### Data Analysis

In order to improve the peak detection by accounting for typical breath-by-breath fluctuations, the VO_2_peak was identified by applying a moving average filter (lag 20, two-sided to avoid phase distortion) to the VO_2_ continuous time-series data. The average for the last five VO_2_ values of fully completed load level (wattage) was used to calculate the participants’ VO_2_peak. (approximately 10 s). We controlled whether participants reached their submaximal performance level at their highest wattage level based on a VO_2_peak >1.5 L/min and a respiratory exchange ratio coefficient (>1.0). The result was considered missing data if these criteria were not met. Missing values (n = 7) were imputed by the expectation-maximization (EM) algorithm.

#### 2.2.3. Driving Task

##### Driving Simulator and Scenario

The driving simulator setup (Carnetsoft version 8.0, Groningen, The Netherlands) consisted of three 48 inch screens laterally angled with a horizontal field of view of 195°, a conventional car seat (VW Golf), a steering wheel and a pedal set (Logitech, International S.A., Lausanne, Switzerland). A traditional numeric keypad with visible numbers from 1 to 6 was located on the right side of the steering wheel, where it could conveniently be reached by participants. The visual field around the screens was covered by black fabric to avoid possible perceptual conflicts with peripheral visual structures. Participants wore a regular headset that was used to present tasks auditorily, and to create a realistic driving sound. More details, and an illustration of the setup, can be found in Wechsler et al. [18]. 

Real-life and laboratory multitasking may differ in their motor, cognitive, and sensorial requirements, leading to a limitation for understanding the driving performance under subtasks [82,83]. Therefore, we used an ecologically valid, virtual driving task that mimics daily car driving, demanding a rich repertoire of motor, cognitive and sensorial actions. The driving scenario consisted of 25.7 km on a rural road, in an environment that included other vehicles, gas stations, traffic signs, mountains, trees, meadows, and small animal enclosures, all under a blue sky with some clouds. The participants’ car was escorted by a rear car that followed the driver at a reasonable distance, and a lead car that drove at 70 km/h. The lead car slowed down slightly if the distance to the participant increased above 100 m, and returned to 70 km/h when the participant’s car caught up. The participants were instructed to follow the lead car at a reasonable distance.

At ten locations along the road, a 40 km/h speed limit sign or a construction site was encountered. There, the lead car performed a braking maneuver: it slowed down to 40 km/h for about 6 s, and then accelerated back to 70 km/h again. These braking sections did not overlap with the tasks of interest for the present study (see “Additional tasks” below) and were not analyzed here. In case participants had an accident, a shattering of the front window was simulated, and the driver’s car was relocated between the rear car and the lead car.

##### Additional Tasks

Participants performed different additional tasks in the driving simulator, a typing task and a reasoning task, both of which were presented either visually or auditorily. 

The typing task mimicked, for example, using a GPS navigator, radio, or bluetooth mobile phone connection. Participants were instructed to type a three-digit number, which was presented via the headset or on the simulated windshield. Digits ranged from one to six and had to be entered into the numeric keypad. The presentation time of the visual stimuli was 5 s, and the presentation time of the auditory stimuli lasted ~3 s. The reasoning task mimicked a conversation with a passenger. It included arguments that could not be answered by “yes” or “no”, and participants were asked to provide verbal answers preferably with one sentence. For example, they were asked to give a reason for cost-free train travel in Germany. Arguments were limited to 10 words and 80 characters. The presentation time of the visual stimuli was 5 s, and the presentation time of the auditory stimuli varied from 3 to 4 s according to the length of the argument. 

All additional tasks were presented in mixed order at irregular time intervals (M = 17.75 s, SD = 4.55 s). Each participant received the same order of tasks at the same locations. No given type of task was presented more than twice in a row in the same modality. In the first phase of the project, additional memorizing tasks were included, where subjects had to compare traffic news (auditory) or gas station prices (visual). As those tasks were not part of the second phase of the project, they were not analyzed here. Nevertheless, the total number of stimuli was similar between both phases (n = 60), while the number of trials per task type and modality differed slightly (n = 10 trials per task type and modality for the first phase of the project and n = 15 trials per task type and modality for the second phase). For further details, see Stojan et al. [73]. 

Participants were instructed to respond to each additional task as quickly and as correctly as possible, and not to prioritize driving or the additional tasks. They were familiarized with the driving simulator for 3–4 min, each for driving only and for the additional tasks only, but not for the combination of both (see Stojan et al. [73] for further information). Familiarization was followed by data registration under three conditions (randomized order): driving only, additional tasks only, or driving while performing the additional tasks. Each driving course took about 25 min. For further details, see Wechsler et al. [18] and Stojan et al. [73]. Here, we analyzed only data of the driving course where participants drove and performed additional tasks. 

##### Data Analysis

Driving performance parameters were recorded continuously at 10 Hz, including the lateral position of the driver’s car and its forward velocity, which are the main outcome parameters of the present study. Both variables are prevalent in driving research and were demonstrated to be responsive to driving under natural conditions and sensitive to distractions through additional tasks performed while driving [18,73,84,85]. These outcome measures were calculated separately for each trial. After outlier rejection by the ±3.29 SD criterion (within each subject and per task type and modality) [86], data were averaged across all trials of a given task type and presentation modality.

The present study addresses driving behavior only. For an analysis of additional task performance, see Wechsler et al. [18].

### 2.3. Procedure

**Figure 1:**
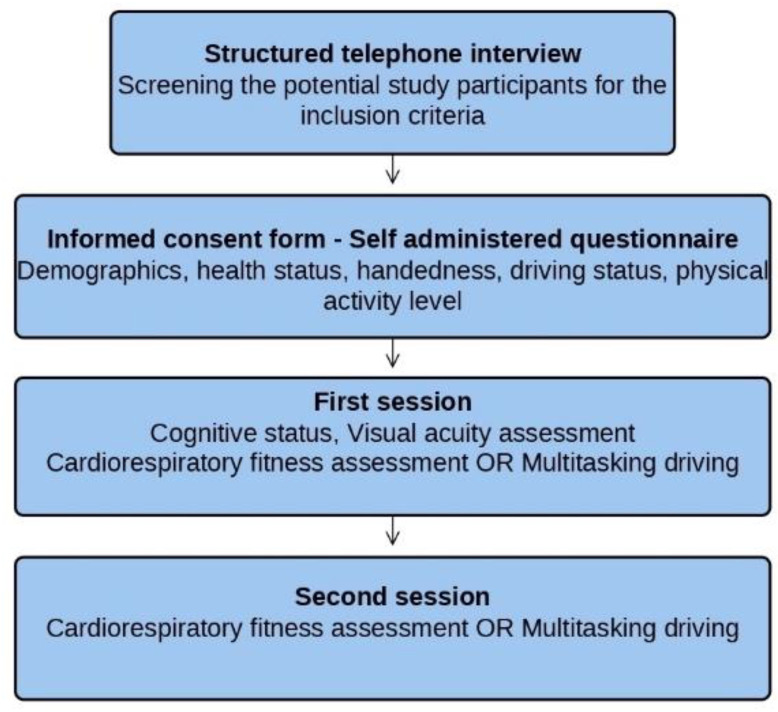
Experiment flow chart.

Before the first session of the experiment, eligible participants determined by telephone interview took general information about the project, informed consent forms, and a questionnaire on demographics, health status, handedness, driving status, and physical activity level via post. The participants were requested to fill out the documents and bring them in the first session. Testing sessions comprised four days in project phase I, and two days in phase II. Because the experimental process included different cognitive and motor tests presented in previous studies [18,73], the order of tests followed pseudorandomized schedules. For this reason, the cardiorespiratory fitness test and multitasking driving task were applied on different days, with at least 24 h off in-between. The experimental flow is depicted in Figure 1.

### 2.4. Statistical Procedures

The first hypothesis on age differences in driving performance was assessed by multivariate analyses of covariance (MANCOVAs), with the dependent variables Mean Velocity and SD Lateral Position separately for all modalities (visual, auditory) of additional tasks (typing, reasoning). Age group (young, young-old, old-old) was added as independent variables. Sex (male/female) and BMI were included as covariates based on the possible influence of these characteristics on the variables of interest [87,88]. To address the second hypothesis, physical activity level (MET) and CRF (VO_2_peak) were entered as continuous variables into the model. As reported above, data were screened for missing values and outliers before the main analysis. They were also screened for violations of MANCOVA assumptions before the main analysis. If the homogeneity of the variance-covariance matrices was violated, Pillai’s Trace value was used to control for biases [86,89]. In case a main or interaction effect reached statistical significance, follow-up univariate analysis of covariance (ANCOVA) were performed for each driving parameter. Pairwise comparisons between groups were performed using estimated marginal means and adjusted through Bonferroni correction. Partial eta squared (*η_p_*^2^) provided an index of effect size [90]. The significance level was set at 0.05. IBM SPSS Statistics, version 25 (IBM Corp., Armonk, NY, USA) was used for these calculations.

## 3. Results

MANCOVA revealed a significant main effect of age on driving performance after controlling for BMI and sex (*p* < 0.001). No significant interaction between age and physical activity level was found. The interaction between age and VO_2_peak on driving performance was significant (*p* = 0.012). MANCOVA results are presented in Table 2.

Univariate follow-up tests for the main effect of age were significant on Velocity and Lateral Position scores for all loading tasks and modalities (Table 3). Pairwise comparisons on the main effects of age on each driving performance variable are presented in Table 4.

**Figure 2:**
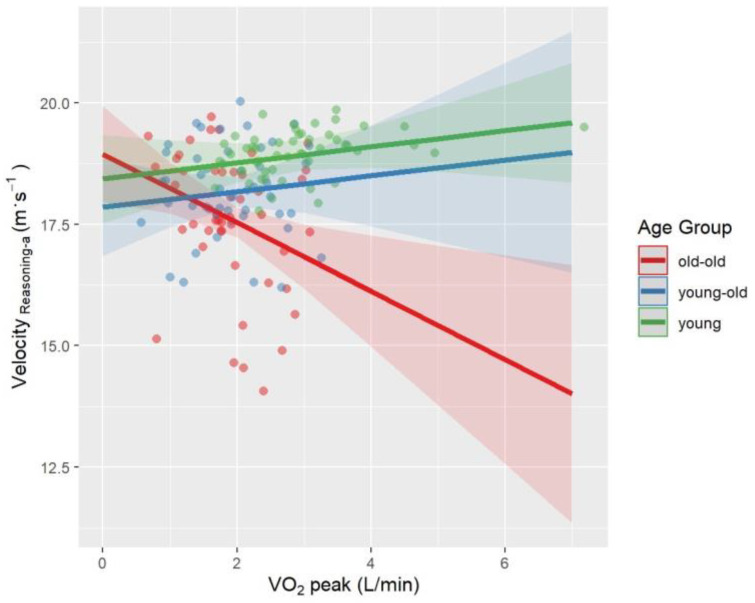
Interaction effect between age and VO_2_peak on Velocity_Reasoning-a_ after controlling for BMI and sex. The fit lines represent the trend of the data for subgroups. Green: young adults, Blue: young-old adults, Red: old-old adults. Young and young-old adults with a higher VO_2_peak drove faster, while old-old adults drove slower.

**Figure 3:**
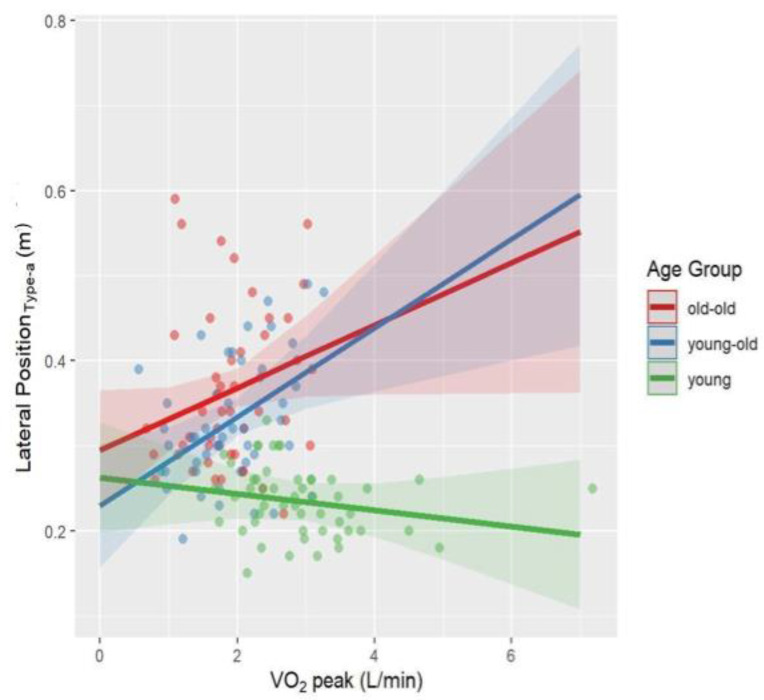
Interaction effect between age and VO_2_peak on Lateral Position_Type-a_ after controlling for BMI and sex. The fit lines represent the trend of the data for subgroups. Green: young adults, Blue: young-old adults, Red: old-old adults. Young adults with higher VO_2_peak drove less laterally, while old-old and young-old adults drove more laterally.

**Figure 4:**
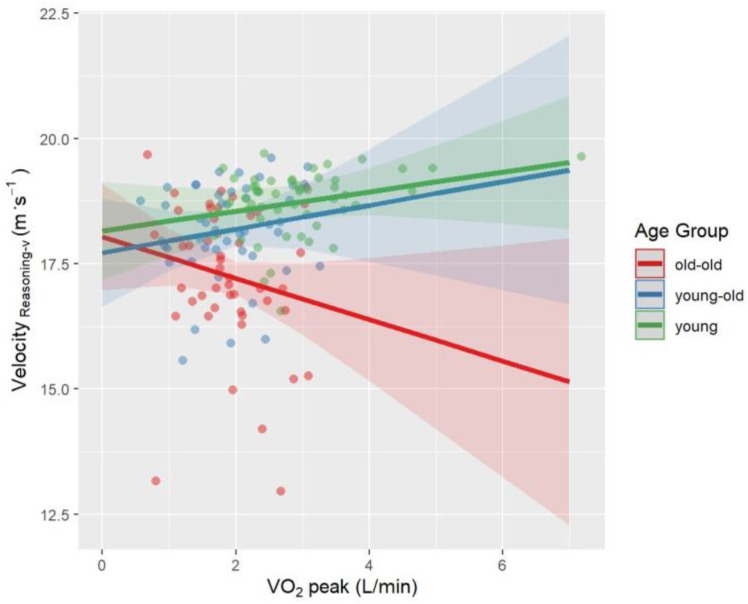
Interaction effect between age and VO_2_peak on Velocity_Reasoning-v_ after controlling for BMI and sex. The fit lines represent the trend of the data for subgroups. Green: young adults, Blue: young-old adults, Red: old-old adults. Young and young-old adults with higher VO_2_peak drove faster, while old-old adults drove slower.

**Figure 5:**
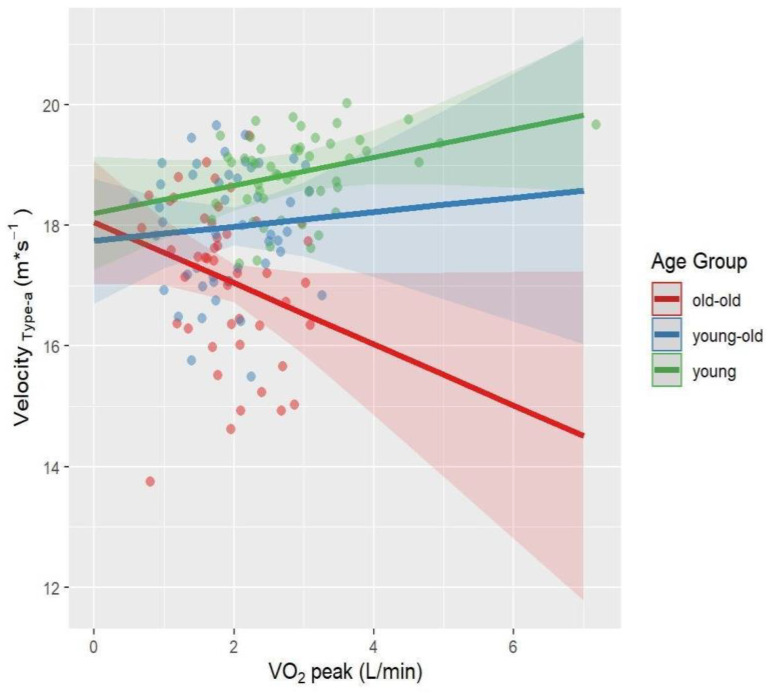
Interaction effect between age and VO_2_peak on Velocity_Type-a_ after controlling for BMI and sex. The fit lines represent the trend of the data for subgroups. Green: young adults, Blue: young-old adults, Red: old-old adults. Young and young-old adults drove faster with higher VO_2_peak, while old-old adults drove slower.

Univariate follow-up tests showed a significant age × VO_2_peak interaction for Velocity_Reasoning-a_ (*p* = 0.03, *η_p_*^2^ = 0.06) and Lateral Position_Type-a_ (*p* = 0.006, *η_p_*^2^ = 0.08). The interaction between age and VO_2_peak approached significance for Velocity_Type-a_ (*p* = 0.05, *η_p_^2^* = 0.04) and Velocity_Reasoning-v_ (*p* = 0.06, *η_p_*^2^ = 0.04). Interaction effects were visualized in Figure 2, Figure 3, Figure 4 and Figure 5. A higher VO_2_peak corresponded to driving faster in young and young-old adults, unlike old-old adults (Figure 2, Figure 4 and Figure 5). A higher VO_2_peak corresponded to driving more laterally (more on the left side of the street) in older adults, unlike young adults (Figure 3).

## 4. Discussion

This study investigated whether advancing age has a deteriorating effect on driving performance and whether physical activity/CRF moderate that hypothesized aging effect. Young, young-old, and old-old adults operated a driving simulator while performing concurrent cognitively demanding tasks in an ecologically valid scenario. Old-old adults drove slower with a more lateral lane position than young ones across the whole driving course. In accordance with our first hypothesis, we also observed differences in driving performance between old-old and young-old adults. Old-old adults drove slower than young-old ones across the whole driving course. Young-old adults drove more laterally across the whole driving course than young ones. They drove slower than young drivers except for the reasoning tasks. Contrary to our second hypothesis, physical activity level did not interact with the aging effect on driving performance. However, cardiovascular fitness interacted with age for the Velocity_Reasoning-a_ and Lateral Position_Type-a_.

### 4.1. Aging Effect

In line with the current literature, our findings showed a detrimental effect of advancing age on driving performance [32,34,91,92]. Research on driving performance demonstrated that advancing age is associated with slower driving speed [18,31,93]. Musselwhite and Haddad [94] reported that older drivers had difficulty maintaining a constant speed at the speed limit, particularly while comprehending road signs, and they compensated for this by driving at low speed. They also showed that reacting accurately when something unpredicted occurred on the road was difficult for older drivers, and a compensation mechanism was again to lower the driving speed. 

Different cognitive, physiological, and perceptual declines have been described and related to diminished driving performance in older adults [71]. Since less cognitive flexibility makes older adults more vulnerable to an unpredictable and changing environment, they may need more time to react correctly. It has also been reported that older drivers tend to brake harder and slower [95,96], and their reaction time was longer to take over the control of the vehicle [97]. Therefore, they may reduce their speed while making driving decisions. Further, older adults’ cognitive processing speed is slower [98], so they might be staring at the speedometer longer than young ones. Besides, accommodation of the eye takes longer in older adults [99]. The difficulty they experience while shifting their gaze between the speedometer and the road might cause them to drive slower as a built-in strategy. Thus, they do not to check the speedometer constantly. Overt orienting of attention (gaze shifting required to control the speedometer) is another cognitive dimension that is known to decline with age [100]. Precise motor control and tactile sense (pressure applied to the gas pedal) are components of the sensorimotor system which are sensitive to age-related deterioration [101,102]. Increased sensory attenuation [103], prolonged movement times, and more corrective sub-movements demand for proprioceptive acuity [104] might lead to poorer speed control in older adults. Whilst the current study did not assess the mentioned cognitive or sensory-motor performances, it is possible to assume that decreased driving ability in old adults might be related to age-related loss in the above-mentioned areas.

Besides confirming earlier findings, our study provided new insights by reporting a gradual decrement in driving speed between young-old and old-old drivers. Data from several sources have identified a gradual decline in processing speed [38,105], cognitive flexibility [38], and visuospatial attentional skills [106,107,108] in older adults. Therefore, our findings might be related to a progressive loss in driving-related cognitive skills with advancing age. In addition, considering that driving slower serves as compensation to cope with decreased cognitive skills, it is likely that old-old adults need more compensation due to their further loss.

Interestingly, there was no significant speed difference between young-old and young adults in the reasoning tasks. The reasoning task may have been less challenging than the typing task as the participants did not need to take their eyes off the screen or perform a manual task to conduct the task, so driving speed may not have differed between young-old and young adults. Another explanation might be that older adults use different cognitive strategies than younger adults to accomplish the argumentation task. Older adults have higher crystallized intelligence and may find it easier to provide arguments to issues of general interest from their higher repertoire of potential answers [92]. Younger adults instead may find this task more difficult due to their lower crystallized intelligence and experience that they can rely on. However, they can still maintain driving performance on a higher level as their brain and cognitive reserve are sufficiently high.

Consistent with what has been found in previous research, our findings demonstrated poorer performance in lane keeping with advancing age [28,109,110,111]. For a safe driving experience, drivers need to perceive, update, inhibit and integrate the information of the environment through attentional control [112] and working memory mechanisms [113,114] continuously. In particular, lane keeping during cognitively demanding driving conditions is particularly related to attentional control and visuospatial working memory [28,115,116,117] which are known to deteriorate with advancing age [118,119,120]. The observed more lateralized driving in the elderly groups could be attributed to the probable decline in the above-mentioned cognitive skills. Unlike our expectations, old-old and young-old adults showed similar lane keeping abilities. It has been proposed that driving at a slower speed reduces the driver’s lateral position [121]. Hence, no significant difference between the old-old and young-old group’s lateral position might be due to the reduced driving speed of the old-old participants. Driving slower might have obscured the difference in lane keeping among the old-old and young-old adults.

Additionally, in our experimental setup, there was a leading car that the driver was constantly following and with which he/she should not cause collision. Furthermore, only a few vehicles came across on the opposite lane. One possible explanation for why driving speed is more susceptible to aging than lane keeping is that speed control might be a more critical parameter to avoid collisions, and old-old adults might have tried to act more controlled in this regard. It should be also noted that multitasking driving studies have shown that driving speed, reaction time, and hazard perception are more susceptible to performance costs than lane keeping [122,123,124]. However, further experimental investigations are needed to understand which driving parameter is more susceptible to demanding tasks and for what reason among older adults.

### 4.2. Interaction Effect of Physical Activity/Cardiorespiratory Fitness and Advancing Age on Driving Performance

Contrary to our expectation, physical activity level did not interact with the advancing age effect on driving performance. Physical activity has been linked to better physical functioning in older adults [125], such as better neck rotation, shoulder flexibility, etc., all of which are associated with good driving behavior [46,126]. Research also indicates that being physically active is associated with generally better brain health and might dampen the age-related deterioration in cognitive skills related to driving, such as processing speed, inhibition, cognitive flexibility, working memory, and attentional orientation [65,127,128,129]. However, our results did not confirm that a higher physical activity level is associated with higher driving behavior, especially for older adults, those more fragile to demanding task conditions. Given the challenging and complex nature of driving, it is likely that the devastating effect of age on driving performance might resist the improving impact of being physically active. It should also be kept in mind that our study population was healthy and physically active adults. Similar physical activity levels of the older age groups may have obscured the potential effect of physical activity on driving performance. A broader range of physical activity levels would provide a more reliable sample to investigate the interaction between advancing age and physical activity level.

While PA level is a behavioral parameter, CRF is described as the capacity of the muscular, respiratory, and circulatory systems to provide oxygen during physical exercise [130]. It should be remembered that although PA level and CRF are related, they are not identical. In this study, contrary to physical activity, CRF interacted with the age effect in Velocity_Reasoning-a_ and Lateral Position_Type-a_. Interaction effect was trend-level for Velocity_Reasoning-v_, Velocity_Type-a_ parameters. Higher-fitness young-old and young adults drove faster while higher-fitness old-old adults drove slower compared to the less fit ones. Higher-fitness young-old and old-old adults drove more laterally, unlike higher-fitness young adults. 

Previous literature on CRF as a predictor of multitasking has discrepancies. Madden and colleagues [131] reported that 16 weeks of aerobic exercise that increases CRF did not improve dual tasking. Dupuy and colleagues [132] demonstrated that higher-fitness middle-aged and older athletes had better dual-task performance than lower-fitness counterparts. A previous path analysis by our team showed that CRF had a trend-level indirect effect on driving speed through cognitive functioning in healthy older adults [73]. This study goes beyond the previous literature by comparing the interaction of age and CRF across different age groups recruiting young, young-old, and old-old adults. As we expected, higher-fitness young-old and young adults drove faster than old-old ones. Our results are also consistent with previous finding that showed that CRF is more associated with driving speed rather than lane keeping in old adults [73]. However, unlike our hypothesis, higher VO_2_peak corresponded to driving slower in old-old adults. We can only speculate about the reasons. The higher-fitness older adults might prefer walking or jogging rather than driving in their daily lives. Another interpretation of this finding might be that the benefit of being a more experienced driver due to chronological age may have covered up the potential positive effect of CRF on driving performance. Yet, driving experience should specifically be evaluated to test these ideas.

Another explanation might be that higher-fitness old-old adults correctly judge their reduced driving skills and therefore reduce their speed as a compensatory measure whereas unfit ones overestimate their driving skills and therefore don’t reduce their driving speed. Given that higher-fitness older adults drove more laterally during the auditory typing task, this unexpected result also may be related to higher-fitness older adults being more likely to engage in risky driving behavior because they are more self-confident. Nonetheless, it is difficult to explain such results without having a more detailed approach that compares the driving habits of the participants. 

Interestingly, the interaction effect of age and CRF on driving performance seems to be related to the stimulus type. This effect was more pronounced in the auditorily presented tasks than visually presented ones in our study. It is widely known that simultaneous performance of two or more different tasks results in the deterioration in task performance and it is referred to as task interference [133]. The interference effect during multitasking might depend on the pairings of input and output modalities [134]. A natural tendency has been proposed to central processing of specific pairings of input and output modalities easier [135]. According to this view, binding auditory stimuli to vocal responses and visual stimuli to manual responses is the preferred processing way due to these pairing being ‘modality compatible’ and characterized by low processing demand [136]. In this case, visual reasoning and auditory typing tasks are incompatible pairings, while auditory reasoning and visual typing are compatible pairings. Given that the interaction effect of age and CRF was more prominent in incompatible pairings in our findings including trend-level results, these tasks might be more susceptible to the supporting effect of higher CRF on driving performance because they are more cognitively demanding. However, further research is required to address different factors influencing the interaction of CRF and advancing age on driving performance, particularly for different age groups.

The present study has some limitations that should be considered when interpreting results. The first limitation is regarding the age ranges of older adults. In our study, the old-old adults were between 70 and 79 years of age. Considering that the population tends to age and life expectancy increases [137], future studies should include the elderly over 80 years old as a third group of older adults.

The second limitation is regarding the driving experience. We questioned the participants’ driving experience in the last six months. However, the information on driving experience across the life span might also be beneficial. These data would enable the assessment of driving performance by controlling the driving experience as much as possible. Future studies should control the driving experience by a more comprehensive assessment.

Finally, because our study consisted of two phases with different numbers of experimental sessions and different durations, this difference varied the durations between the assessment of VO_2_peak and the driving task. In future studies, the time between two sessions should be standardized.

## 5. Conclusions

As far as we know, no previous research has addressed the potential discontinuity in advancing age effects on driving speed and lane keeping during multitasking driving. 

We believe that investigating different groups of older adults could contribute to understanding the development of driving performance within older adults.

The present study showed that age-related driving performance varied in the young-old and old-old adults, and performance losses are not identical for the entire elderly phase. These findings highlight a critical point for aging studies and suggest that the typical results obtained for the young-old adults cannot be generalized to the old-old ones. At a more general level, understanding the development course may guide planning therapeutic strategies for the requirements of older adults on time. Our data suggest that past research on older drivers is difficult to interpret because the age range in those earlier studies either was very wide or was not specified. Future work should clearly distinguish between the performance of young-old and old-old drivers.

For the first time, we demonstrated that a high cardiovascular fitness could contribute to good driving performance in young and young-old adults, unlike old-old adults. Future studies are needed to get a deeper insight into the interaction of physical activity level/CRF and advancing age on driving performance in different age groups. In this way, the potential contribution of physical activity to provide and maintain a safe driving experience can be evaluated more comprehensively.

## Figures and Tables

**Table 1:** Demographic data.

Variable	Young (n = 48)	Young-Old (n = 47)	Old-Old (n = 46)	*p*
Age	23.25 ± 2.82	66.53 ± 1.50	74.21 ± 2.33	<0.001 **
Sex (m|f)	19|29	25|22	29|17	<0.001 **
BMI (kg/m^2^)	22.24 ± 2.36	25.19 ± 2.90	25.31 ± 2.89	<0.001 **
Education (years)	15.43 ± 2.36	15.65 ± 2.69	15.83 ± 3.35	0.680
MET (min/week)	5.92 ± 1.33	5.31 ± 1.33	5.87 ± 1.93	0.076
VO_2_peak (L/min)	2.97 ± 0.95	1.90 ± 0.65	1.89 ± 0.62	<0.001

Note: Means and standard deviations are presented. BMI = Body mass index, MET = metabolic equivalent, VO_2_peak = peak oxygen consumption. One-way ANOVA and Chi-squared test were performed to analyze the group differences. ** *p* < 0.001.

**Table 2:** MANCOVA results.

	Pillai’s Trace	*F*	d*f*	*p*	*η_p_* ^2^
Age	0.639	7.628	16, 260	<0.001 **	0.32
PA level	0.054	0.919	8, 128	0.49	0.05
VO_2_peak	0.049	0.830	8, 128	0.51	0.05
Age × PA level	0.126	1.039	24, 381	0.38	0.06
Age × VO_2_peak	0.307	1.807	24, 381	0.012 *	0.10

Note: Age included three levels (young, young-old, old-old). PA level and VO_2_peak were continuous independent variables. Gender and BMI were included as covariates. * *p* < 0.05 ** *p* < 0.001.

**Table 3:** Univariate follow-up results for the main effect of age.

Driving Parameter	Young	Young-Old	Old-Old	*F* ^b^	df	*p*	*η_p_* ^2^
Velocity_Type-v_	19.09 (0.16)	18.11 (0.15) ^Y^	17.37 (0.15) ^Y,YO^	26.10	2	<0.001 **	0.27
Velocity_Type-a_	18.87 (0.16)	17.96 (0.15) ^Y^	17.13 (0.15) ^Y,YO^	26.99	2	<0.001 **	0.28
Velocity_Reasoning-v_	18.74 (0.16)	18.19 (0.15)	17.25 (0.16) ^Y,YO^	19.35	2	<0.001 **	0.22
Velocity_Reasoning-a_	18.88 (0.16)	18.23 (0.15)	17.65 (0.15) ^Y,YO^	14.03	2	<0.001 **	0.17
Lateral Position_Type-v_	0.21 (0.008)	0.29 (0.008) ^Y^	0.30 (0.008) ^Y^	29.50	2	<0.001 **	0.30
Lateral Position_Type-a_	0.23 (0.012)	0.32 (0.011) ^Y^	0.36 (0.011) ^Y^	29.23	2	<0.001 **	0.30
Lateral Position_Reasoning-v_	0.18 (0.007)	0.22 (0.006) ^Y^	0.22 (0.006) ^Y^	8.60	2	<0.001 **	0.11
Lateral Position_Reasoning-a_	0.19 (0.006)	0.23 (0.006) ^Y^	0.23 (0.006) ^Y^	11.18	2	<0.001 **	0.14

Note: Type: typing, visually (-v) and auditorily (-a), ^Y^: young, ^YO^: young-old. ^Y,YO^ indicate the denoted value is significantly different from that of the group in the same column. Data are presented as mean ± standard deviation. ** *p* < 0.001. ^b^ ANCOVA with sex and BMI as covariates.

**Table 4:** Pair-wise comparisons for the effect of age on each driving performance variable score between age groups.

Driving Parameter	Old-Old vs. Young-Old		Old-Old vs. Young		Young-Old vs. Young	
	t	*p*	t	*p*	t	*p*
Velocity_Type-v_	3.41	0.003 *	7.21	<0.001 **	4.21	<0.001 **
Velocity_Type-a_	3.84	0.001 *	7.33	<0.001 **	3.92	<0.001 **
Velocity_Reasoning-v_	4.18	<0.001 **	6.02	<0.001 **	2.29	0.071
Velocity_Reasoning-a_	2.75	0.020 *	5.28	<0.001 **	2.84	0.051
Lateral Position_Type-v_	0.90	0.364	7.41	<0.001 **	5.75	<0.001 **
Lateral Position_Type-a_	2.26	0.096	7.41	<0.001 **	5.75	<0.001 **
Lateral Position_Reasoning-v_	0.33	0.758	4.00	0.001 *	3.66	0.001 *
Lateral Position_Reasoning-a_	0.01	0.997	4.33	<0.001 **	4.33	<0.001 **

Note: Type: typing, visually (-v) and auditorily (-a). * *p* < 005, ** *p* < 0.001.

## Data Availability

Data are available upon request from the corresponding author.
